# A Favourable and Singular Termination of the Hæmorrhoids

**Published:** 1809-12

**Authors:** George Cope

**Affiliations:** Surgeon, Leek, Staffordshire


### *A favourable and singular Termination of the Hæmorrhoids, by* GEORGE COPE, *Surgeon, Leek, Staffordshire.*

*Hæmorrhois, a Genus in the class Pyrexiæ, and order Hæmorrhagia,—Berkenhout.*

I Was desired to visit a young man of a strong, robust, athletic habit, much addicted to drinking, and other irregularities, severely afflicted with the hæmorrhoids, internally and externally. He had taken before I saw him Sal. Glauber ℥ij ; had no sleep for two nights and two days, was thirsty, hot, restless, and full of pain; he took an anodyne draught at bed time, and passed a tolerable night; next day leeches were applied, but without any apparent relief; the draught repeated at bed-time, with the following emollient anodyne fomentation.

℞. Capit. papav. incis. No. vi. Folior malvæ. Flor. Sambucin a Miij. coq. in. Aq. font. ℔iv. ad. ℔iij. cola et liquori colato, adde Extr. Saturn. (Goulard) ℥iss. M. ft. fotus sæpissime in die applic.

The next day was much easier, though no sensible diminution in the volume of the protrusion; the draught was repeated at bed time, and his bowels kept moderately open with Ol. Ricin. interposing occasionally a grain or two of calomel; and in order to supply the place of the fomentation, which could not be so conveniently applied during the night, the annexed ointment was directed.

℞. Ung. Saturn flav. Ung. ex Althæâ. Ung. Sambucin a ʒj. Camphor. Pulv. opii a ʒß. M. ft. Ung. quocum pars affecta omni nocte h. s. bene inung. The draught repeated, and the fotus continued during the day. In the dead of the night, he was awoke with an insupportable pain in the left groin ; and on examination, the inguinal glands were found tense, hard, much enlarged, and covered with a considerable efflorescence; the hæmorrhoids at the same time exhibiting a livid gangrenous aspect. An adhesive plaster was immediately applied to the left groin, in order to forward maturation, and hasten suppuration, which was accomplished in 36 hours from the attack, the tumour bursting spontaneously with a profuse discharge. In addition to the extraordinary novelty of the case, we found the hæmorrhoids decrease, in proportion as the inguinal tumour increased, till not a vestige could be traced. This may be considered a critical abscess which nature (per Metastesin) employed to unload the morbid hæmorrhoidal congestion, congestion, or it may be said to be a conversion of one disease into another.

Humani natura corporis custos fidelis est. It appears from the writings of Hippocrates, Galen, Celsus, Hildanus, &c. that the hæmorrhoids are conducive to health when kept within proper bounds; but a suppression, diminution, or excess may produce Apoplexy, Epilepsy, Vertigo, Inflammation of the Eyes, Aphony, Phthisis Pulmonalis, Dropsy,* Tabes, Fistulous Ulcers, Cancers, and innumerable other diseases intimately connected with the seat, use, and structure of the different viscera. As Hippocrates has wisely observed,

Ὅλος ἄνθρωπος ἐκ γενετῆς νόσος ἐστι.

It is with pleasure I add, that the patient's health is completely re-established, having no return of the complaint, now seven months since) to which he is subject on the commission of any irregularity in living.

* Si ora venarum sanguinem solita fundere, subito, suppressa sunt, aut aqua inter cutem, aut tabes sequitur.—CELSUS Lib. 2, Cap. vij.

